# Abortion Following Removal of a Cervical Polypus

**Published:** 1887-03

**Authors:** W. H. Lancaster

**Affiliations:** Coleman, Texas


### ABORTION FOLLOWING REMOVAL OF A CERVICAL POLYPUS.

*By W. H. Lancaster, M. D,, Coleman, Texas.*

For Daniels Texas Medical Journal.

ON September 1, 1886, Mr. D. called at my office asking me to prescribe for his wife, adding that he had tried two other doctors, but that their prescriptions had failed to give any relief.

After saying to him that if he did not allow me a better opportunity than he had the other doctors, I did not want to attempt to do anything, I obtained the following. His wife complained of soreness of lower bowels and abdomen. Especially the right side; was nervous, having hot flashes, spells of faintness and full feeling in the head. Soreness and pain in the right side constant, but worse at the time of her periods, and aggravated by being on her feet. Had missed her period. Bowels constipated. Abdomen would swell up at times, which would interfere with her breathing. When the bowels would swell and puff up there was very considerable oppression, and a feeling as though she were loosing her mind. Appetite good at times; at times none. Never vomitted; was never sick, or nauseated at all. We demurred to the husband at making such random prescriptions without an examination, and suggested to him that the reproductive system was at fault, and the prime etiological factor in her case, and that, though I would not refuse to prescribe for his wife, I did not expect such treatment to benefit her much, if at all. He then stated that both the other doctors had referred him to the reproductive system for her trouble and one of them insisted upon treating her for uterine disease, but they refused him. Hereupon he asked me to call down to see his wife in person. This I did and obtained the following history: Mrs. D. was 27 years old. Had been married five years. Had never been pregnant. Was never regular in her life with monthly periods. Had at times missed as much as five months successively. Had been treated for uterine troubles by two physicians before coming to this place. By one for one year continuously, but not later than two or three years. She always suffered with dysmenorrhœa, and the flow was very scanty; suffered with much pain and soreness at such times. This originated by being on the feet much. Had missed her show last month and it was now time, as she could best judge, for her menses again, but no show; had heavy, bearing down sensation in womb and pelvis. Was not allowed any examination of the womb. Prescribed a tonic and digestive; advised the hot vaginal douche twice daily, and stated that unless further notified or solicited I would dismiss the case.

On September 13th Mr. D. called again, saying the prescription had done no good and asking me to take charge of the case properly. I at once proceeded to mke a digital examination, which revealed a slightly enlarged uterus, cervix a little large and elongated, with something quite filling up the cavity of the cervix and on a level with the lips. This caused me to further interrogate the parties and to examine the breasts. This examination of the mammæ failed to offer any such sign as to me indicated pregnancy. I introduced the speculum. This revealed cellular polypus, quite fllling up the cervical canal. It was greatly compressed, and presented a double teat-like tumor or substance no longer than the cervix, merely showing between the lips. Although, as said, a cellular, rather vascular structure, it was quite devoid of mucus or blood; indeed rather dry and friable. Further manipulation proved it to be attached to the left lateral cervical wall, just outside of the os internum. The nutrition was considerably interfered with. I called the husband's attention to the existence of the tumor; stated the propriety of removing it at once, and as soon as I could step to my office and procure a pair of forceps, by torsion with slight traction, I removed it without difficulty. This being completed, I stated to Mr. D. that the womb was too large, and that it was possible that another polypus existed, as I thought the one removed not sufficient, attached as it was, to account for the increase in size of the uterus (and, though we had no hemorrhage, an almost constant symptom of polypus). I could not decide what caused the enlargement, if not intra-uterine polypus, and added that the presence, situation and shape of the one removed so fully and perfectly filled up the cervical canal as to preclude, to my mind, the possibility of pregnancy. After applying Tr. Iodine (Churchill's) to the stump of the polypus, I advised continuance of the vaginal hot douche. Prescribed F. E. Ergot & Gossip: Rad: to be taken three times daily, hoping thereby to expel other polypi, I left to call for a week later. On the 18th returned, found the condition quite as before. No flow; womb apparently larger. I again examined the breasts, but did not decide that such changes existed as indicated pregnancy. I again introduced the speculum, and with a sound and dilator made a thorough search for other polypi, but found none. Did not note any change in the cervix that was not attributable to the existence of the polypus; applied iodine to cervix. On the 25th called again; size of womb increased. Again examined breasts; stated that I did not know how to account for size of uterus except by pregnancy. There has never existed the least nausea; rests well at night; continued hot douches. Called again October 1st; refused to make application to the cervix; state that I am quite convinced now that pregnancy exists. Womb is still larger. Mrs. D. denies that the mammæ are larger than usual; they are not at all tender; no secretion. Advised her to go about her work as usual, or as though she were well. State positively that she is pregnant, which very much displeased the lady. On 29th find case progressing nicely; development quite apace with the supposed time of gestation. State in substance that they may not need a doctor for five or six months; ask to be advised if all is not well. Heard no more of the case until November 19th, when upon my return from the country, where I had been detained for a couple of days, I learned that on the morning of the 18th Mr. D. had called for me and that the call was answered by my partner, Dr. Bowers, who stated that he found the womb trying to expel its contents and that it were better to allow it to go on, as there was an offensive odor with the discharge; said there was something in the womb that should pass out. Charged to watch for any hemorrhage and report at once, if any. During the night of the 18th because of a free discharge which Mr. and Mrs. D. supposed was blood, Dr. Bowers was again called, but found it to be amniotic fluid and quited their fears. On the 19th, upon my return, Dr. Bowers stated the above. He and I at once visited the case; the family were again informed that the contents of the womb must and would pass away; there were then no pains, scarcely, but the os was partially dilated. On the night of the 19th Mrs. D. was delivered of a dead fœtus of about 3 or 3½ months. Apropos to this case please allow it said, that the first time I made a digital examination Mrs. D. remarked that she expected I would find her different from any woman I ever examined, and as a reason, was told that her doctor who had treated her formerly said so, and also said she could never give birth to a child. My opinion was asked and was given as follows: That it would be with difficulty and great suffering that she could give birth to a full-term, well developed child. The question directed my attention especially to the contour and size of the pelvis. I found the pelvis small, with the deepest and most acute angle to the pubic arch I ever saw. I did not attempt to take measurements. Now as very severe criticisms have been passed upon this case and the management thereof, both by some professional brethren and by members of the family, both male and female busy bodies, and as Mr. D. has been urged to prosecute and sue me, and very many false statements have been made relative to the case, we ask the publication of these facts. Morever we ask full and free criticism. We feel quite justifiable under the existing circumstances for excluding pregnancy at first, yet assert that we have good and sufficient authority for the removal of the polypus, though we were later convinced that pregnancy did exist. Now first, as to the exclusion of pregnancy; the woman was never regular, always had a very scanty flow, had frequently missed several periods in succession, at one time seeing no show for so long a time as five months. Had been married five years without having conceived. Would be classed as a masculine type of woman. Had suffered much with phenomena referable to the reproductive system. Had previously been treated for uterine disease not, however, within two years time or more. Absence of every sign of pregnancy except menstrual cessation; was just then time for the second menstrual mollumen since show was seen. Please note that at first a rigid attempt was made to ascertain pregnancy. Secondly, the existence of the polypus, "one of the effects of which is sterility," this one so especially, situated as to perfectly occlude the cervical canal. It was so compressed by the uterus as to almost cut off all blood supply. Was not this sufficient, ordinarily to prevent the advent of spermatozoa into the womb, and is it not quite consistent with reason to suppose that it had done so in this instance, for times past? We ask the question: Were not the very facts surrounding and existing in this case sufficient to justify the exclusion of pregnancy? And had we been convinced that pregnancy did exist were we not justifiable in the removal of the polypus? The search in the womb for another polypus was only justifiable upon the exclusion of pregnancy. Resume: The existence of the polypus might and does produce sterility, and increase in the size of the womb through the attendant hyperæmia and later, hyperplasia, dysmenorrhœa, irritation and the attendant discomfort, and in fact any and every symptom. The sympathetic digestive and nervous phenomena, etc., unless it be excepted the menstrual cessation, and as by mechanical obstruction it may, and does, produce dysmenorrhœa, may it not further, acting as a ball valve, absolutely obstruct the cervical canal?

